# Fusion imaging in brain structure measurements on a fetus phantom, combining real‐time ultrasound with magnetic resonance imaging

**DOI:** 10.1002/ajum.12246

**Published:** 2021-05-25

**Authors:** Anastasija Arechvo, Göran Lingman, Lars Thurn, Tomas Jansson, Ligita Jokubkiene

**Affiliations:** ^1^ Department of Obstetrics and Gynecology Skåne University Hospital Lund University Lund Sweden; ^2^ Department Obstetrics and Gynecology IKVL Medical Faculty Lund University Lund Sweden; ^3^ Department of Clinical Sciences Lund Biomedical Engineering Lund University Lund Sweden; ^4^ Clinical Engineering Skåne Medical Services Lund Sweden

**Keywords:** ultrasound, MRI, fusion imaging, fetal brain, phantom

## Abstract

**Objectives:**

To assess synchronisation of MRI and US in measuring foetus phantom head structures; inter‐method, intra‐ and inter‐observer differences on biparietal diameter (BPD), head diameter, anterio‐posterior head diameter (HAP) and lateral ventricle structures (VS).

**Methods:**

Fusion Imaging (FI) has been performed by combining MRI and US simultaneously. Axial scans of 1.5 Tesla MRI on a foetus phantom were acquired and uploaded on a US machine (EPIQ 7G, Philips). A PercuNav US tracker allowed the system to recognise and display the position of the transducer. A fetal phantom tracker was used as a phantom reference. Real‐time US of the phantom head was performed by synchronising the uploaded MRI images using different landmarks. Synchronisation has been assessed by taking measurements after rotating the US probe by 90. Measurements were taken by three different observers twice. Differences in measurements between MRI and US, inter‐, intra‐observer differences in all measurements were assessed.

**Results:**

BPD, HAP and VS measurements before rotation were 0.13 ± 0.06 cm, 0.46 ± 0.09 cm and 0.4 ± 0.23 cm (width) and mean 0.6 ± 0.25 cm (length) larger at MRI than at US using any number of landmarks. After US probe rotation VS were 0.3 ± 0.24 cm in width and 0.3 ± 0.27 cm in length. Intra‐ and inter‐observer differences in all measurements were small.

**Conclusions:**

FI showed good synchronisation in measurements. BPD, HAP and VS were larger at MRI than US, likely a result of the way images are generated. Intra‐, inter‐observer differences between measurements were small. This can be important when reporting geometric measures from FI.

## Introduction

The use of magnetic resonance imaging (MRI) in combination with real‐time ultrasound (US) has been recently introduced in medical fields such as neurosurgery and urology.^1^, ^2^ It has also been used in detecting deep infiltrating endometriosis.^3^ MRI and real‐time US simultaneous imaging, called fusion imaging (FI), has been observed to be feasible and potentially helpful in improving even prenatal assessment of fetal malformations such as cerebral malformations or lung lesions.^4^

Real‐time US remains the method of choice for screening and evaluation of fetal malformations, however, in cases of abnormal findings, MRI is used as a complementary diagnostic method.^5^, ^6^ MRI has an excellent soft‐tissue resolution, which allows detailed visualisation of fetal cerebral structures; however, ultrafast T2 MRI sequences enable good imaging quality by the minimised effect of fetal movements.^4^, ^6^ Diagnostic accuracy in detecting small fetal brain structures might be improved by combining MRI and real‐time US simultaneously.

Despite FI as a combination of MRI and real‐time US in prenatal fetal brain assessment has been used in some studies,^4^ no studies describing the methodology for simultaneous use of US and MRI could be found. In a previous study by Sarris et al. the intra‐ and inter‐observer variability in fetal ultrasound measurements were discussed.^7^ In a study comparing US and MRI in the assessment of fetal biometry and weight it was shown that both techniques are operator dependent and are subject to random error.^8^ However, inter‐method, inter‐ and intra‐observer variability comparing simultaneous measurements from combined MRI and US imaging have not been previously assessed.

In earlier studies, measurements of fetal BPD and lateral ventricles have been found to be larger with MRI than US.^6^, ^9^ Therefore, before estimating the benefits of the FI technique in fetal brain structure measurements and assessment, methodological studies are needed.

The aim of our study was to assess the synchronisation of real‐time MRI and US on a fetus phantom in measuring structures imitating fetal head and brain by using different numbers of matching landmarks, and to assess inter‐method, intra‐observer and inter‐observer differences in fetus phantom head measurements (biparietal diameter (BPD), head diameter (HD), anterio‐posterior head (HAP) diameter and lateral ventricle structures (VS)).

## Materials and methods

This is a prospective study. Two ultrasound prenatal specialists and one ultrasound trainee performed the measurements in the study. The study object was a biometric fetal ultrasound training phantom (CIRS, Virginia, USA) corresponding to an average human fetus at gestational week 21. The fetus phantom was made of proprietary gels with a speed of sound of 1540 m/s while the housing was made of polyvinyl chloride (PVC), acrylonitrile butadiene styrene (ABS) and vinyl with a speed of sound at 1550 m/s. Bones of the fetus phantom had the same speed of sound as the fetus at 1540 m/s and ventricles at 1550 m/s.

**Figure 1:**
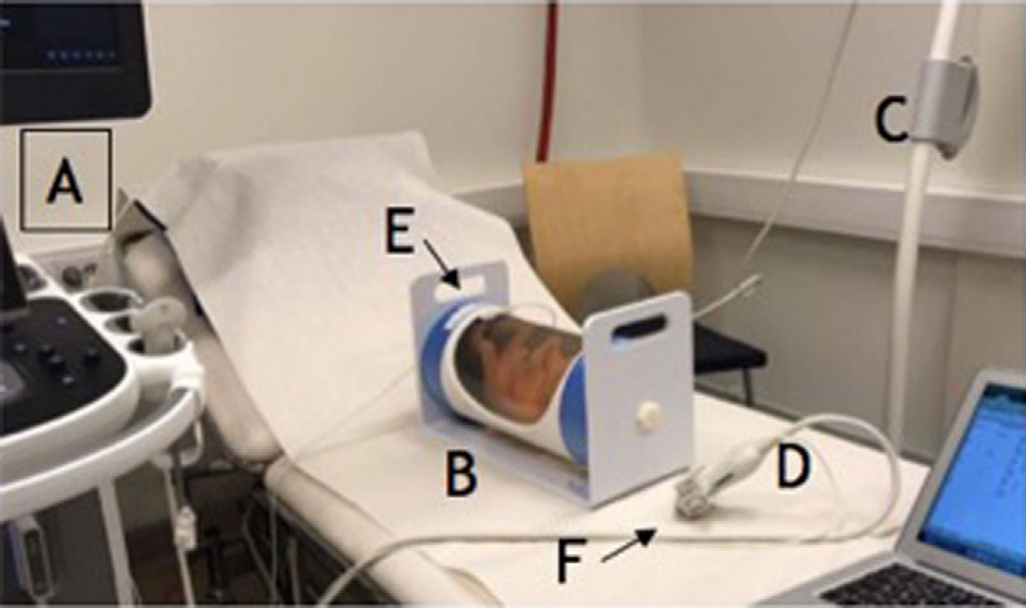
The experimental tools needed to perform fusion imaging. A: US machine. B: Fetal US training phantom. C: Magnetic field transmitter. D: US probe. E: Phantom tracker. F: PercuNav US tracker. US (ultrasound), MRI (magnetic resonance imaging).

The MRI examination of the fetal phantom was performed using 1.5 Tesla MRI (Philips, Netherlands) prior to the US examination. Axial MRI scans (1 mm slices) were then uploaded on the US machine (EPIQ 7G, Philips, Netherlands) using DICOM^®^ (Digital Imaging Communications in Medicine, USA) datasets. The ultrasound transducer (C9‐2 Curved Ultrasound Probe, Philips, Netherlands) was connected to the navigation system, PercuNav Software (Philips, Netherlands) US tracker, allowing the system to recognise and display the position and orientation of the transducer. During US scanning, the magnetic tracking system determined the position and orientation of the movable PercuNav US tracker that was fixed to the US probe, as well as the position of a special fetal phantom tracker, used as a phantom reference (Figure 1). All measurements have been made on the same ultrasound machine by three observers independently as shown in Figure 6.

**Figure 2:**
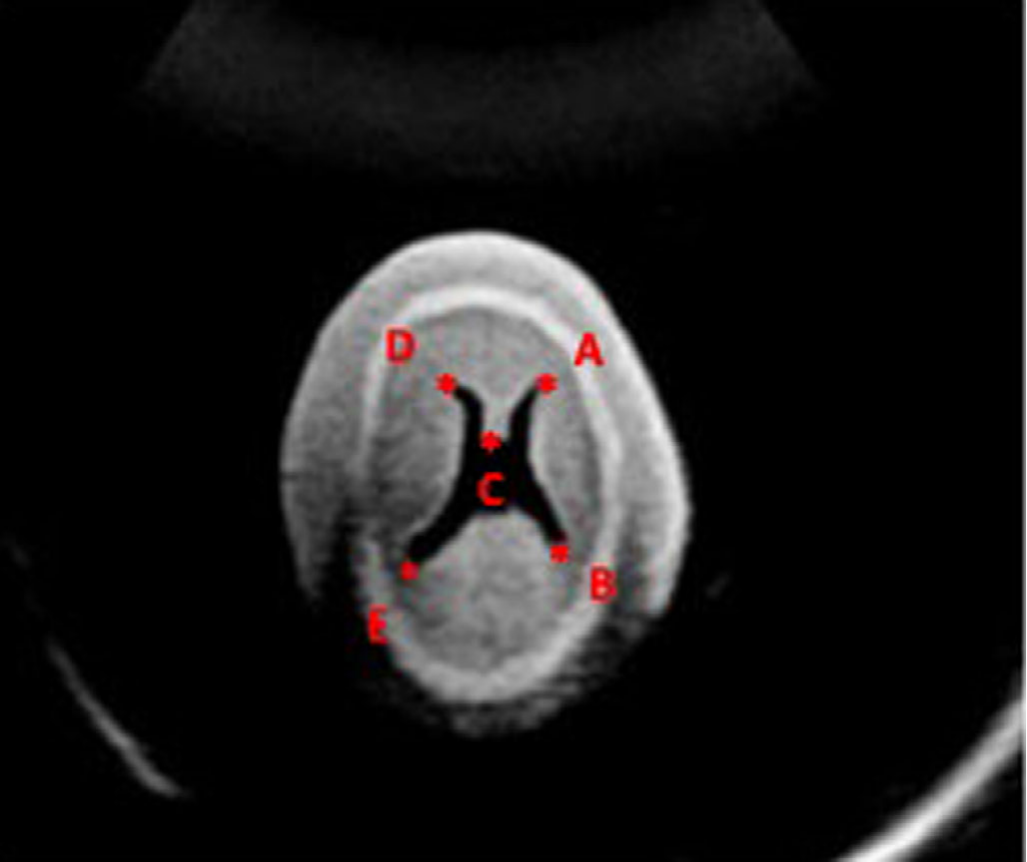
Placement of the landmarks. One, three, four and five landmarks. A: when one landmark is used; ABC: when three landmarks are used; ABCD: when four landmarks are used; ABCDE: when five landmarks are used.

**Figure 3:**
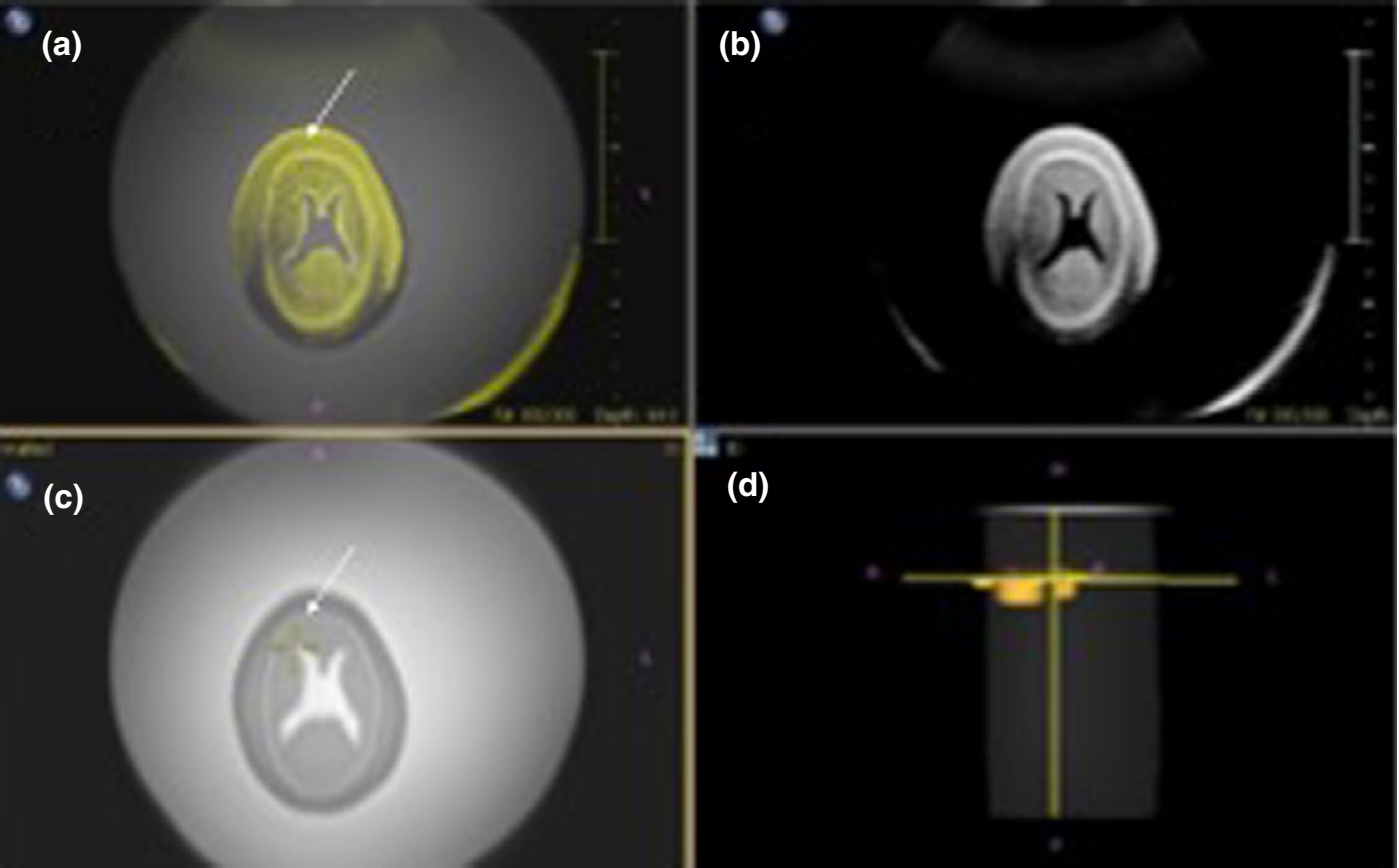
Image showing axial plane of fetal phantom head before the US probe rotation with 90°. A: Fused image of real‐time US and MRI. B: Real‐time US image. C: MRI image. D: projection of US probe. The “+” is showing a location of internal landmark. US (ultrasound), MRI, magnetic resonance imaging; BPD, biparietal diameter, HD, head diameter; HAP, anterio‐posterior head diameter; VS, lateral ventricle structures.

Movement and position data were transmitted to the US system for the synchronisation with the MRI data. Axial images of the fetal phantom head with MRI and ultrasound at the level in the ventricular system were identified. To visualise the two modalities simultaneously, matching landmarks were used finding similar structures (reference points) on both MRI and US images separately on the axial image. One, three, four and five matching landmarks were used and placed by each observer independently after agreement on the number of markers and definitions on the measurements have been made by all three observers. Placement of the markers is shown in Figure 2. The fused axial MRI‐US images were then displayed simultaneously side‐by‐side as well as overlaid atop each other (Figure 3).

**Figure 4:**
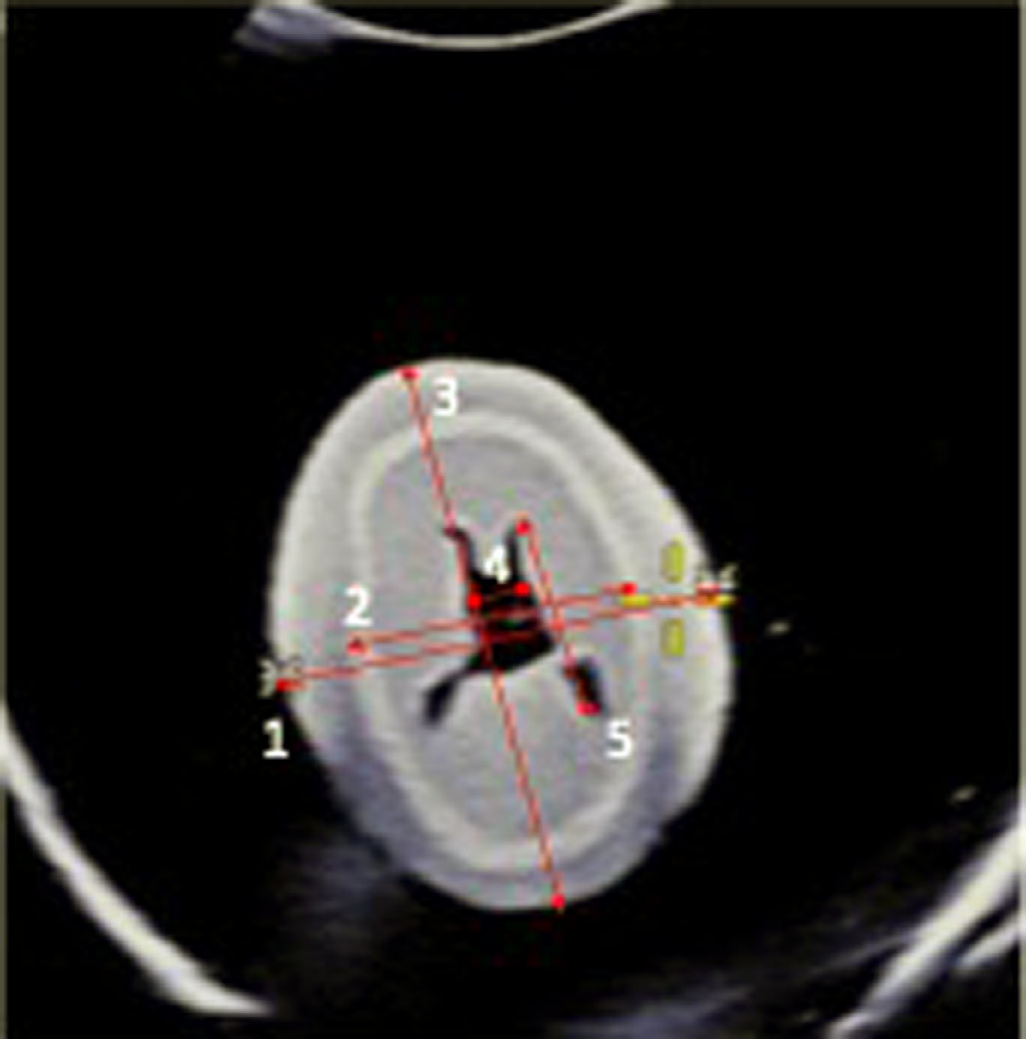
Image showing measurements taken on fetal phantom on axial plane. BPD, HD, HAP, VS width and length. 1: BPD measurement was taken as a distance between biparietal (outer‐outer) bones; 2: HD as a transverse distance between scull surface (outer‐outer); 3: Anterio‐posterior head (HAP) diameter (or occipito‐frontal distance (outer‐outer); 4: VS width (outer‐outer); 5: VS length (outer‐outer). US (ultrasound), MRI, magnetic resonance imaging; BPD, biparietal diameter; HD, head diameter; HAP, anterio‐posterior head diameter; VS, lateral ventricle structures.

Measurements of phantom fetal head structures (Figure 4) – BPD, HD and HAP diameter, as well as lateral ventricle measurements, were taken on axial plane T2 MRI and US images separately using one, three, four and five matching landmarks. Due to technical configurations, the fusion imaging system is not allowing to use two landmarks. Following measurements were taken on axial plane: BPD – as a distance between outer to outer contour of biparietal bones; HD – as a transverse distance between outer to outer contour of the phantom head soft tissues; anterio‐posterior head (HAP) diameter as outer to outer contour of the phantom head soft tissues; width and length of lateral ventricles as inner to inner contour of the ventricles shown at Figure 4. Both anterio‐posterior and transverse measurements of the structures of different size were chosen keeping in mind possible effect on measurements of the ultrasound beam and the MRI technique. Measurements of all structures were compared between MRI and US. The only raw measurement provided by the manufacturer was BPD. Measurements of the BPD were compared with reference measurement provided by the manufacturer of the fetal phantom with a target biometric BPD of 4.2 cm.

**Figure 5:**
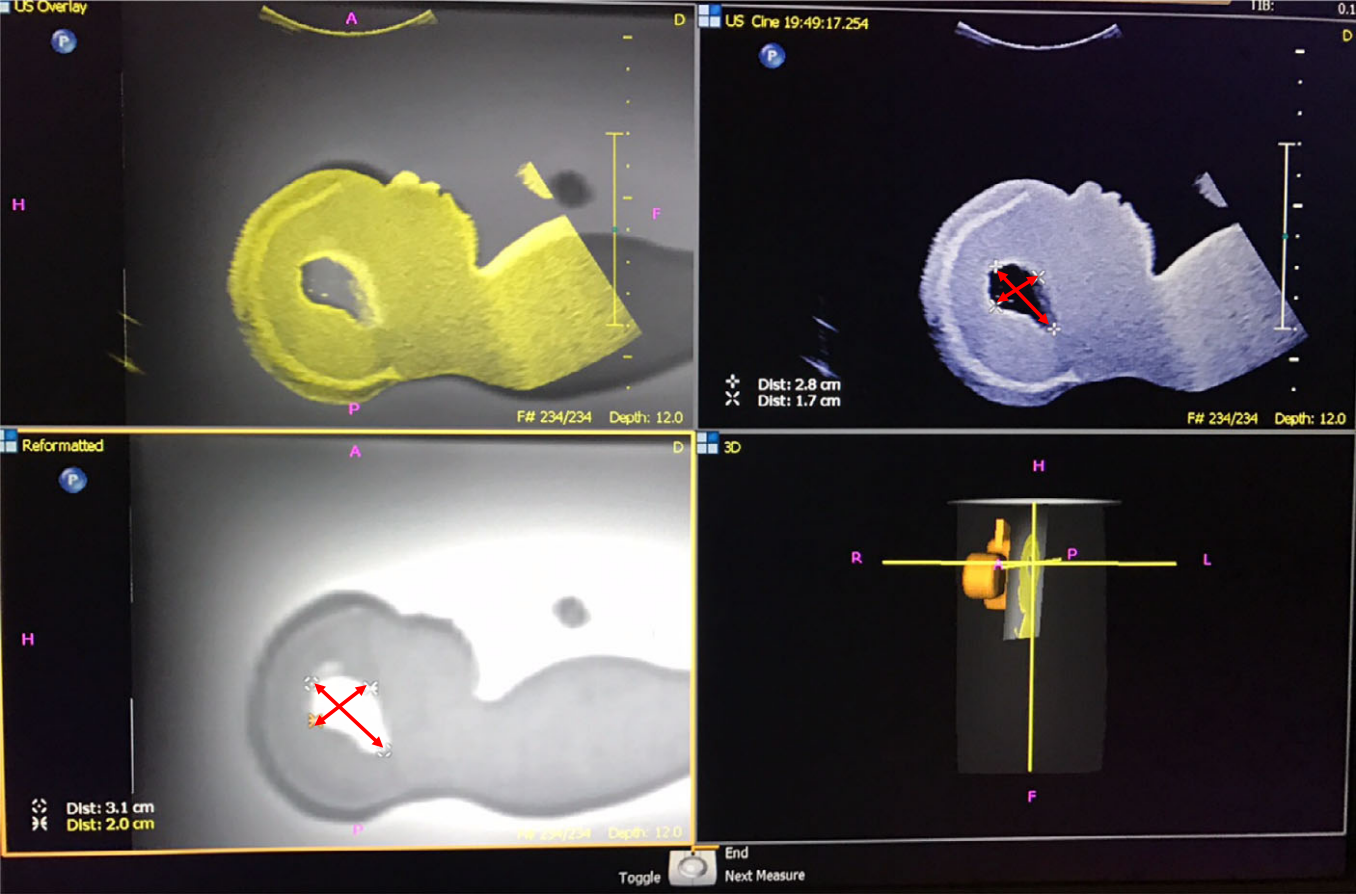
Image showing the sagittal plane of the fetal phantom head after US probe rotation of 90°. A: Fused image of real‐time US and MRI. B: Real‐time US image. C: MRI image. D: projection of US probe. US (ultrasound), MRI, magnetic resonance imaging. Arrows shows measurements taken on fetal phantom sagittal plane.

After measurements were taken on the axial plane, the ultrasound probe was rotated 90° to assess synchronisation of US and MRI. As MRI and US were fused by using matching landmarks on axial images, MRI image rotated simultaneously with US image. Images on the sagittal plane of MRI and US were acquired. Lateral ventricle structures were measured by taking width and length after the US probe rotation with 90° to the sagittal plane (Figure 5).

To assess synchronisation, lateral ventricle measurements on MRI and US were compared. It has been repeated using one, three, four and five matching landmarks. Small or no differences in lateral ventricle measurements between MRI and US were considered being good synchronisation.

**Figure 6:**
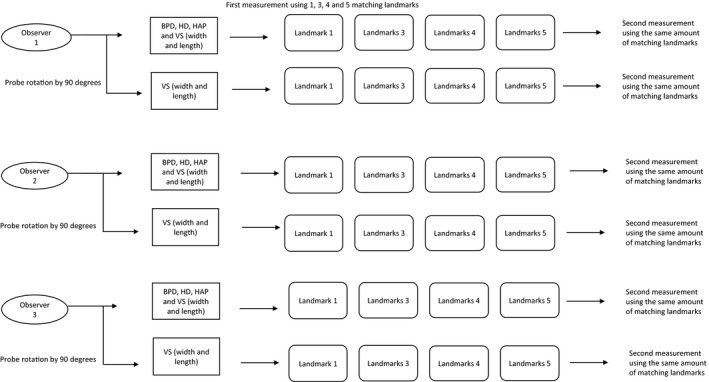
The sequence of measurements by the different operators. Measurements were taken both on ultrasound and magnetic resonance imaging images twice by every observer. BPD, biparietal diameter; HD, head diameter; HAP, anterio‐posterior head diameter; VS, lateral ventricle structures.

Each examiner performed all measurements independently twice as shown in Figure 4. Observers had predetermined placement of matching landmarks. Measurements were taken blinded and independent of measurements taken by other observers. The sequence of measurements by the different operators is shown in Figure 6. Differences in measurements within each observer (intra‐observer differences) and between measurements between different observers (inter‐observer differences) were calculated for three observer pairs.

### Statistical analysis

Statistical calculations were performed using Statistical Package for the Social Sciences (SPSS Inc., Chicago, IL, USA) version 25. The sample size analysis was not performed. Differences between all measurements were calculated comparing measurements taken with MRI and US. Differences of the first measurements in hands of all observers of anatomical structures (biparietal diameter, head diameter, anterio‐posterior head, and length and lateral ventricle structures) were calculated. BPD measurements with MRI and US were compared with the given manufacturer measurement. Mean difference and 95% CI was calculated. If 95% CI range included zero, it was considered to be no difference between measurements. Differences in measurements within each observer (intra‐observer difference) and between first and second measurements among different observers (inter‐observer difference) were calculated for three observer pairs. Differences for intra‐observer are presented together for all landmarks, as measurements between measurements using one, three, four and five landmarks were small.

### Ethical approval

Not required.

## Results

Mean differences between first measurements at MRI and US using a different number of matching landmarks are shown in Table 1. BPD and ventricle structures measurements were larger with MRI than US despite the number of landmarks used. HD was smaller measured with MRI than US using one or three landmarks, but no difference was found in measurements using four or five landmarks. No differences were found in HAP measurements using any number of landmarks.

**Table 1:** Inter‐method differences. Comparison between MRI and US in first measurements of BPD, HD, HAP and lateral ventricle width and length measurements (cm) before US probe rotation with 90° in the hands of three observers and difference between measurements (MRI minus US) by using different number of landmarks. Measurements for MRI and US are given as median (range). Bold typeface is used where the bias is larger than the standard deviation.

Number of landmarks	MRI, cm	US, cm	Difference (cm), mean, ± SD	95% CI
BPD				
1	3.6 (3.6–3.8)	3.5 (3.4–3.6)	0.13, ±0.08	0.05–0.22
3	3.9 (3.8–3.9)	3.8 (3.6–3.8)	0.13, ±0.05	0.08–0.12
4	3.9 (3.8–4.0)	3.8 (3.7–3.9)	0.10, ±0.06	0.03–0.17
5	4.0 (3.8–4.0)	3.8 (3.7–3.9)	0.15, ±0.06	0.09–0.21
HD				
1	5.6 (5.4–5.6)	5.7 (5.6–5.8)	−0.13, ±0.12	−0.26 to −0.01
3	5.6 (5.5–5.6)	5.7 (5.6–5.8)	−0.10, ±0.06	−0.17 to −0.04
4	5.6 (5.4–5.6)	5.7 (5.4–5.7)	**−0.08**, ±0.12	−0.21–0.04
5	5.6 (5.5–5.7)	5.7 (5.6–5.9)	**−0.10**, ±0.11	−0.21–0.02
HAP				
1	7.3 (7.2–7.6)	7.3 (7.2–7.4)	**0.03**, ±0.09	−0.05–0.12
3	7.4 (7.3–7.4)	7.3 (7.3–7.4)	**0.03**, ±0.05	−0.02–0.09
4	7.4 (7.2–7.5)	7.3 (7.2–7.4)	**0.07**, ±0.08	−0.02–0.15
5	7.4 (7.2–7.5)	7.4 (7.2–7.4)	**0.05**, ±0.15	−0.10–0.21
VS width				
1	2.6 (2.4–3.1)	2.3 (2.0–2.4)	0.43, ±0.27	0.15–0.71
3	2.6 (2.5–2.8)	2.3 (2.0–2.4)	0.37, ±0.23	0.13–0.60
4	2.7 (2.4–2.8)	2.4 (1.7–2.4)	0.48, ±0.22	0.25–0.72
5	2.8 (2.6–2.9)	2.3 (1.8–2.7)	0.50, ±0.24	0.25–0.74
VS length				
1	3.2 (2.8–3.6)	2.6 (2.4–2.8)	0.57, ±0.31	0.24–0.90
3	3.4 (3.1–3.6)	2.8 (2.6–3.0)	0.53, ±0.18	0.35–0.72
4	3.4 (3.2–3.5)	2.7 (2.4–2.9)	0.70, ±0.18	0.51–0.90
5	3.3 (3.2–3.5)	2.5 (2.2–2.8)	0.80, ±0.25	0.53–1.07

US, ultrasound; MRI, magnetic resonance imaging; BPD, biparietal diameter; HD, head diameter; HAP, anterio‐posterior head diameter; VS, lateral ventricle structures; CI, confidence interval; SD, standard deviation.

BPD varied between 3.6–4.0 cm at MRI and 3.4–3.9 cm at US when measured by different observers. BPD at MRI was 0.13 cm (±0.06) (mean (±SD)) larger than those at US images and was larger despite the number of landmarks used. BPD measured with US and MRI differed and was smaller than biometric BPD measurement, by 0.4 cm on MRI and 0.5 cm on US.

The head diameter varied between 5.4–5.9 cm at US and 5.4–5.7 cm at MRI, while the mean difference in HD between all observers between US and MRI was found to be −0.1 cm (±SD (±0.09)).

Anterior‐posterior head diameter (HAP) varied between 7.2–7.4 cm in US images and 7.2–7.6 cm in MRI with a mean difference of 0.46 cm (±SD (±0.09)) and were larger in MRI images than in US images irrespective of the number of landmarks used.

The size of the lateral ventricle varied between 1.7–2.7 cm in width at US and 2.4–3.1 cm at MRI and 2.2–3.0 cm and 2.8–3.6 cm in length, respectively, and these measurements were mean 0.4 cm (width) (±SD (±0.23)) and mean 0.6 cm (length) (±SD (±0.25)) larger measured with MRI than with ultrasound.

After the US probe rotation by 90° to assess synchronisation of US and MRI, the measurements of the lateral ventricle varied between 1.1–1.8 cm in width and 2.0–3.2 cm in length with US and between 1.2–2.1 cm in width and 2.6–3.7 cm in length with MRI. Measurements were larger with MRI than US by 0.3 cm (±0.24) (mean (±SD)) in width and 0.3 cm in length (0.27) (mean (±SD)). That was true for a different number of landmarks and in the hands of all observers for all measurements but VS width using three markers and VS length using one landmark (Table 2).

**Table 2:** Measurements of lateral ventricle (width and length) after US probe rotation with 90° and difference between measurements at MRI and US by using one, three, four and five landmarks to assess synchronisation. Measurements for MRI and US are given as median (range). Bold typeface is used where the bias is larger than the standard deviation.

Number of landmarks	MRI, cm	US, cm	Difference, (cm) mean, ±SD	95% CI
VS width				
1	1.9 (1.8–2.1)	1.6 (1.4–1.8)	0.32, ±0.23	0.07–0.56
3	2.0 (1.2–2.1)	1.8 (1.1–1.8)	**0.22**, ±0.41	−0.22–0.65
4	2.0 (1.7–2.1)	1.7 (1.3–1.8)	0.33, ±0.12	0.21–0.46
5	1.8 (1.6–2.2)	1.6 (1.4–1.7)	0.28, ±0.13	0.14–0.42
VS length				
1	2.9 (2.6–3.5)	2.8 (2.4–3.0)	**0.18**, ±0.31	−0.14–0.50
3	3.3 (2.8–3.7)	2.8 (2.1–3.2)	0.47, ±0.27	0.18–0.75
4	3.0 (2.9–3.4)	2.8 (2.7–2.9)	0.25, ±0.23	0.00–0.50
5	3.0 (2.8–3.2)	2.8 (2.0–2.9)	0.33, ±0.27	0.05–0.61

US, ultrasound; MRI, magnetic resonance imaging; VS, lateral ventricle structures; CI, confidence interval; SD, standard deviation.

Three different observers performed all measurements twice. Differences in measurements on US and MRI between observers are shown in Table 3. Differences in all measurements between observers (inter‐observer difference) were small, with a mean (±SD) difference of −0.09 (±0.94) cm (95% CI −0.13–‐0.05) on MRI and −0.05 (±0.12) cm (95% CI −0.11–0.00) on US.

**Table 3:** Inter‐observer means differences (median (range)) in measuring BPD (biparietal diameter), HD (head diameter), HAP (anterio‐posterior head diameter), VS (lateral ventricle structures) width and length in cm. Bold typeface is used where the bias is larger than the standard deviation.

Measurement	Observer 1 – Observer 3 mean, ±SD	95% CI	Observer 1 – Observer 2 mean, ±SD	95% CI	Observer 2 – Observer 3 mean, ±SD	95% CI
Inter‐observer (Difference between measurements between different observers on US for different number of landmarks and all landmarks together)						
BPD						
One	0.00, 0.00	0.00–0.00	**0.10**, 0.14	−1.17–1.37	**−0.10,** 0.14	−1.37–1.17
Three	**0.05**, 0.07	−0.58–0.68	**0.15**, 0.07	−0.48–0.78	−0.10, 0.00	−0.10 to −0.10
Four	0.00, 0.14	−1.27–1.27	0.00, 0.00	0.00–0.00	0.00, 0.14	−1.27–1.27
Five	**−0.05**, 0.07	−0.68–0.58	**0.05**, 0.07	−0.58–0.58	**−0.10**, 0.14	−1.37–1.17
All landmarks	0.00, 0.75	−0.06–0.06	0.07, 0.08	0.00–0.14	**−0.07**, 0.10	−0.16–0.01
HD						
One	−0.10, 0.00	−0.10 to −0.10	**0.05**, 0.07	−0.58–0.68	**−0.15**, 0.07	−0.78–0.48
Three	−0.10, 0.00	−0.10 to −0.10	0.00, 0.00	0.00–0.00	−0.10, 0.00	−0.10 to −0.10
Four	**−0.05**, 0.07	−0.68–0.58	**0.15**, 0.07	−0.48–0.78	**−0.20**, 0.14	−1.47–1.07
Five	**−0.20**, 0.14	−1.47–1.07	−0.10, 0.00	−0.10 to −0.10	**−0.10**, 0.14	−1.37–1.17
All landmarks	−0.11, 0.08	−0.18 to −0.04	**−0.02**, 0.10	−0.06 – 0.11	−0.13, 0.09	−0.21 to −0.06
HAP						
One	−0.05, 0.21	−1.95–1.85	−0.05, 0.07	−0.68–0.58	−0.10, 0.00	−0.10 to −0.10
Three	−0.05, 0.07	−0.68–0.58	0.00, 0.00	0.00–0.00	−0.05, 0.07	−0.68–0.58
Four	0.00, 0.14	−1.27–1.27	0.05, 0.07	−0.58–0.68	−0.05, 0.07	−0.68–0.58
Five	−0.15, 0.07	−0.78–0.48	0.00, 0.00	0.00–0.00	0.05, 0.21	−1.85–1.95
All landmarks	−0.06, 0.11	−0.16 – 0.03	0.00, 0.05	−0.04–0.04	−0.03, 0.10	−0.12–0.05
VS width						
One	0.00, 0.14	−1.27–1.27	0.20, 0.14	−1.07–1.47	−0.20, 0.28	−2.74–2.34
Three	0.15, 0.21	−1.75–2.05	0.00, 0.00	−1.27–1.27	0.05, 0.21	−1.85–1.95
Four	0.60, 0.14	−0.67–1.87	−0.05, 0.07	−0.68–0.58	0.65, 0.07	0.01–1.28
Five	0.10, 0.42	−3.71–3.91	−0.35, 0.21	−2.25–1.55	0.40, 0.00	0.40–0.40
All landmarks	0.21, 0.31	−0.04–0.47	−0.05, 0.23	−0.24–0.14	0.22, 0.37	−0.08–0.53
VS length						
One	−0.05, 0.21	−1.95–1.85	0.15, 0.21	−1.75–2.05	−0.20, 0.00	−0.20 to −0.20
Three	−0.20, 0.28	−2.74–2.34	−0.15, 0.35	−3.32–3.02	0.05, 0.21	−1.85–1.95
Four	−0.10, 0.28	−2.64–2.44	−0.25, 0.07	−0.88–0.38	0.15, 0.35	−3.02–3.32
Five	−0.10, 0.14	−1.37–1.17	0.20, 0.28	−2.34–2.74	−0.30, 0.42	−4.11–3.51
All landmarks	−0.11, 0.18	−0.27–0.04	−0.01, 0.27	−0.24–0.22	−0.07, 0.29	−0.32–0.17
VS width 90°						
One						
Three	−0.35, 0.07	−0.98–0.28	−0.20, 0.00	−0.20 to −0.20	−0.15, 0.07	−0.78–0.48
Four	−0.30, 0.28	−2.84–2.24	−0.40, 0.42	−4.21–3.41	0.00, 0.00	0.00–0.00
Five	−0.15, 0.35	−3.32–3.02	−0.30, 0.28	−2.84–2.24	0.15, 0.07	−0.48–0.78
All landmarks	−0.20, 0.14	−1.47–1.07	−0.30, 0.00	−0.30 to −0.30	0.10, 0.14	−1.17–1.37
	−0.25, 0.20	−0.41 to −0.08	−0.30, 0.20	−0.47 to −0.12	0.02, 0.13	−0.09–0.14
VS length 90°						
One						
Three	−0.35, 0.21	−2.25–1.55	−0.10, 0.42	−3.91–3.71	−0.25, 0.21	−2.15–1.65
Four	−0.50, 0.14	−1.77–0.77	−0.40, 0.56	−5.48–4.68	−0.40, 0.00	−0.40 to −0.40
Five	−0.15, 0.07	−0.78–0.48	−0.15, 0.07	−0.78–0.48	0.00, 0.00	0.00–0.00
All landmarks	0.40, 0.56	−4.68–5.48	−0.10, 0.14	−1.37–1.17	0.50, 0.42	−3.31–4.31
	−0.15, 0.43	−0.51–0.21	−0.18, 0.30	−0.44–0.06	−0.03, 0.40	−0.37–0.30
Inter−observer (Difference between measurements between different observers on MRI for different number of landmarks and all landmarks together)						
BPD						
One	0.1, 0.14	−1.17–1.37	0.05, 0.21	−1.85–1.95	0.05, 0.07	−0.58–0.68
Three	0.0, 0.00	0.00–0.00	0.10, 0.00	0.10–0.10	−0.10, 0.00	−0.10 to −0.10
Four	−0.05, 0.07	−0.68–0.58	0.05, 0.07	−0.58–0.68	−0.10, 0.14	−1.37–1.17
Five	0.05, 0.07	−0.58–0.68	0.10, 0.14	−1.17–1.37	−0.05, 0.21	−1.95–1.85
All landmarks	0.02, 0.09	−0.05–0.10	0.07, 0.10	−0.01–0.16	−0.05, 0.11	−0.14–0.04
HD						
One	0.00, 0.14	−1.27–1.27	−0.10, 0.14	−1.37–1.17	0.10, 0.00	0.10–0.10
Three	−0.05, 0.07	−0.68–0.58	−0.05, 0.07	−0.68–0.58	0.00, 0.00	0.00–0.00
Four	−0.15, 0.07	−0.78–0.48	−0.10, 0.00	−0.10 to −0.10	−0.05, 0.07	−0.68–0.58
Five	−0.05, 0.07	−0.68–0.58	−0.10, 0.00	−0.10 to −0.10	0.05, 0.07	−0.58–0.68
All landmarks	−0.06, 0.09	−0.13–0.01	−0.08, 0.06	−0.14 to −0.03	0.02, 0.07	−0.03–0.08
HAP						
One	−0.15, 0.07	−0.78–0.48	−0.15, 0.07	−0.78–0.48	0.00, 0.14	−1.27–1.27
Three	−0.05, 0.07	−0.68–0.58	−0.10, 0.00	−0.10 to −0.10	0.05, 0.07	−0.58–0.68
Four	−0.10, 0.28	−2.64–2.44	−0.05, 0.21	−1.95–1.85	−0.05, 0.07	−0.68–0.58
Five	−0.15, 0.21	−2.05–1.75	−0.15, 0.07	−0.78–0.48	0.00, 0.28	−2.54–2.54
All landmarks	−0.11, 0.14	−0.23–0.01	−0.11, 0.09	−0.19 to −0.02	0.00, 0.13	−0.10–0.10
VS width						
One	−0.40, 0.42	−4.21–3.41	0.05, 0.21	−1.85–1.95	−0.45, 0.21	−2.35–1.45
Three	−0.25, 0.07	−0.88–0.38	−0.10, 0.14	−1.37–1.17	−0.15, 0.07	−0.78–0.48
Four	0.25, 0.07	−0.38–0.88	0.05, 0.07	−0.58–0.68	0.20, 0.14	−1.07–1.47
Five	−0.05, 0.21	−1.95–1.85	−0.10, 0.00	−0.10 to −0.10	0.05, 0.21	−1.85–1.95
All landmarks	−0.11, 0.31	−0.37–0.15	−0.02, 0.12	−0.13–0.08	−0.08, 0.29	−0.33–0.15
VS length						
One	−0.70, 0.14	−1.97–0.57	−0.30, 0.00	−0.30 to −0.30	−0.40, 0.14	−1.67–0.87
Three	−0.35, 0.21	−2.25–1.55	−0.25, 0.07	−0.88–0.38	−0.10, 0.28	−2.64–2.44
Four	0.15, 0.07	−0.48–0.78	−0.05, 0.7	−0.68–0.58	0.20, 0.00	0.20–0.20
Five	−0.15, 0.21	−2.05–1.75	−0.20, 0.14	−1.47–1.07	0.05, 0.07	−0.58–0.68
All landmarks	−0.26, 0.35	−0.55–0.33	−0.20, 0.11	−0.29 to −0.10	−0.06, 0.26	−0.28–0.16
VS width 90°						
One	0.10, 0.14	−1.17−1.37	0.20, 0.14	−1.07–1.47	−0.10, 0.00	−0.10 to −0.10
Three	−0.60, 0.42	−4.41–3.21	−0.45, 0.35	−3.62–2.72	−0.15, 0.07	−0.78–0.48
Four	−0.30, 0.14	−1.57–0.97	−0.25, 0.07	−0.88–0.38	−0.05, 0.07	−0.68–0.58
Five	−0.35, 0.21	−2.25–1.55	−0.25, 0.21	−2.15–1.65	−0.10, 0.42	−3.91–3.71
All landmarks	−0.28, 0.33	−0.56 to −0.01	−0.18, 0.30	−0.44–0.06	−0.10, 0.16	−0.24–0.04
VS length 90°						
One	−0.30, 0.56	−5.38–4.78	0.00, 0.14	−1.27–1.27	−0.40, 0.56	−5.48–4.68
Three	−0.55, 0.49	−4.99–3.89	−0.70, 0.00	−0.70 to −0.70	0.15, 0.49	−4.29–4.59
Four	0.20, 0.42	−3.61–4.01	0.10, 0.28	−2.44–2.64	0.10, 0.14	−1.17–1.37
Five	−0.25, 0.07	−0.88–0.38	−0.05, 0.07	−0.68–0.58	0.00, 0.28	−2.54–2.64
All landmarks	−0.22, 0.43	−0.59–0.14	−0.16, 0.35	−0.46–0.13	−0.03, 0.38	−0.35–0.28

US, ultrasound; MRI, magnetic resonance imaging; BPD, biparietal diameter; HD, head diameter; HAP, anterio‐posterior head diameter; VS, lateral ventricle structures; CI, confidence interval; SD, standard deviation.

Differences between two measurements by the same observer (intra‐observer difference) are shown in Table 4. Mean (±SD) intra‐observer difference in all measurements for observer 1 was 0.58 cm (±0.09) for US measurements and −0.01 cm (±0.08) for MRI; for observer 2 was 0.04 cm (±0.18) and 0.14 cm (±0.32) respectively; observer 3 had the mean difference on UL at −0.04 cm (±0.07) and on MRI measurements −0.05 cm (±0.04).

**Table 4:** Intra‐observer differences in measuring BPD (biparietal diameter), HD (head diameter), HAP (anterio‐posterior head diameter), VS (lateral ventricle structures) width and length in cm with one, three, four and five landmarks (all landmarks added).

Measurement	MRI (Mean, ±SD)	95% CI	US (Mean, ±SD)	95% CI
Observer 1				
BPD	0.05, ±0.10	−0.10–0.20	0.02, ±0.05	−0.05–0.10
HD	0.07, ±0.12	−0.12–0.27	0.07, ±0.05	−0.01–0.15
HAP	−0.10, ±0.08	−0.23–0.03	−0.05, ±0.05	−0.14–0.04
VS width	0.05, ±0.17	−0.22–0.32	0.05, ±0.05	−0.04–0.14
VS length	−0.05, ±0.12	−0.25–0.15	0.02, ±0.15	−0.21–0.26
VS width 90°	−0.15, ±0.33	−0.67–0.37	0.05, ±0.05	−0.04–0.14
VS length 90°	0.00, ±0.37	−0.59–0.59	0.25, ±0.36	−0.33–0.83
Observer 2				
BPD	0.05, ±0.05	−0.04–0.14	0.07, ±0.12	−0.12–0.27
HD	0.00, ±0.00	0.00–0−00	0.10, ±0.08	−0.03–0.23
HAP	0.07, ±0.18	−0.22–0.38	0.12, ±0.09	−0.02–0.27
VS width	−0.22, ±0.09	−0.38 to −0.07	−0.02, ±0.35	−0.59–0.54
VS length	−0.07, ±0.20	−0.40–0.25	−0.30, ±0.14	−0.52 to −0.07
VS width 90°	0.75, ±0.22	−0.28–0.43	0.07, ±0.18	−0.22–0.37
VS length 90°	0.40, ±0.35	−0.16–0.96	0.30, ±0.36	−0.28–0.88
Observer 3				
BPD	−0.10, ±0.08	−0.23–0.03	−0.07, ±0.05	−0.15–0.01
HD	0.00, ±0.08	−0.13–0.13	0.07, ±0.09	−0.07–0.22
HAP	−0.02, ±0.12	−0.22–0.17	0.00, ±0.08	−0.13–0.13
VS width	−0.05, ±0.10	−0.20–0.10	−0.17, ±0.12	−0.37–0.02
VS length	−0.10, ±0.14	−0.32–0.12	−0.05, ±0.26	−0.47–0.37
VS width 90°	−0.07, ±0.09	−0.22–0.07	−0.02, ±0.05	−0.10–0.05
VS length 90°	−0.02, ±0.12	−0.22–0.17	−0.07, ±0.26	−0.27–0.12

## Discussion

Our study was performed to assess synchronisation of MRI and US scans on a fetus phantom head by fusion imaging using real‐time US and MRI. Synchronisation of real‐time US and MRI was assessed after rotating the US probe by 90°. We have also assessed differences in measurements on MRI and US for all examined structures using a different number of landmarks at standardised planes in the hands of three observers.

Fusion imaging as a diagnostic technique proposed to be highly relevant in prenatal diagnosis.^10^ Several potential advantages have to be mentioned. Salomon et al. newly described how FI can be used in educational purposes; improving diagnostic capabilities and even proposing improved guiding of invasive procedures during the pregnancy.^11^ However, to the best of our knowledge, this study is the first study that describes the methodology of simultaneous use of US and MRI despite that this technique, fusion imaging, has been used widely in the medical field. The results of our study show that it is possible to obtain accurate synchronisation between images of real‐time US and MRI after US probe rotation by 90°, and this can be achieved by using one, three, four and five matching landmarks when landmarks are placed as described above.

We found that BPD and VS measurement differences were larger with MRI than US when both methods are used simultaneously, while there were no differences in other measurements (HAP and HD using four and five landmarks). The theoretical explanation for these differences could be that measurement size depends on the insonation angle and the fact that insonation angle changes during the examination that can affect measurements, or the way bone and fluid are represented in MRI and US images (see discussion below).

Differences in measurements at MRI and US may cause concern, as discrepancies between MRI and US measurements can lead to incorrect clinical patient counselling, for example in case of fetal ventriculomegaly. Our results are in agreement with a clinical study by Behrendt et al. ​that found that MRI measurements of fetal brain ventricles were significantly larger than the measurements by US by approximately 1 mm.^9^ We did not observe differences in other measurements such as HAP and HD. Parkar et al.^6^ found that there was good agreement in fetal measurements of abdominal diameter, abdominal circumference, head circumference at MRI and US but not BPD. According to authors, the discordance between two modalities could be connected to techniques and imaging technology.

The fact that differences between MRI and real‐time US were observed on some but not all measurements may be explained by MRI and US fundamental differences in image formation: MRI uses the combination of a strong magnetic field and radiofrequency waves and image tissue contrast is influenced by the proton density of tissue, reflecting the water content. On the other hand, ultrasound uses high‐frequency sound waves to form images, where the difference in acoustic impedance between investigated tissue structures give rise to echoes. This means that there is a difference in signal generation between the two modalities: while cerebral fluid contained in the ventricles are represented as black on ultrasound (no internal echoes), it is in white on MRI imaged, reflecting the high water content in the investigated voxel. Larger measurements of lateral ventricles on MRI than US may be explained by differences in signal formation between these two imaging modalities as well. Since an echo, or MRI‐signal, from a boundary to water‐filled cavity is represented by a finite size line or region in an image, signal energy “spills over” into voids where no signal is present. Therefore, ventricles will appear smaller in ultrasound images, and similarly, appear larger in MRI images. Therefore, it is not surprising that there is a mismatch in measurements between MRI and US, MRI showing larger values. The spatial resolution is within the same order of magnitude between the two modalities in this case, even though the spatial resolution is better for ultrasound than MRI (approximately by a factor of 2). In relation to this discussion, this would imply a smaller error compared to the ground truth for ultrasound. However, this difference in spatial resolution between ultrasound and MRI is not expected to have a large impact on the measurements in this study as it falls within the standard deviation of intra‐ and inter‐observer differences in the measurements.

We compared measurements on MRI and US images between three different observers and within the same observer, that is inter‐ and intra‐observer differences. We found differences in all measurements being small between all three observers. There was no systematic bias within each observer in any measurement for neither US nor MRI. Differences between observers in all measurements were small and no systematic bias was observed. However, our study was performed on fetal phantom and further studies on a live fetus are needed.

A study by Perni et al.^12^ on intra‐ and inter‐observer reliability of fetal biometry measurements by ultrasound showed high intra‐ and inter‐observer reproducibility. A comparison between US and MRI on common fetal measurements by Parkar et al.^6^ showed good agreement between US and MRI for head circumference, mean abdominal diameter and abdominal circumference but not for BPD and femur length. Similar study performed by Garel et al.^13^ comparing fetal lateral ventricles on US and MRI showed good agreement between these techniques.

The strength of our study is that we used a fetal phantom in a gel having the same sound speed in all structures similar to human body soft structures of 1540 m/s. Another strength is that this is the first methodological study of fusion of MRI and US imaging, despite that fusion imaging has been used widely in the medical field. We recommend to use one, three, four and five landmarks placed as shown in Figure 4 to achieve synchronisation and a small difference in measurements with no systematic bias.

Our study had several limitations. The main limitation of our study is that we could not compare our measurements on US and MRI with the real size of different phantom structures as only the size of BPD was provided by the manufacturer. However, raw data could still not exclude the possible effect on measurements by insonation angle or differences in imaging techniques. Another limitation is that only a limited number of structures could be measured, and therefore it was not possible to assess the possible effect of insonation angle on additional measurements. It might be considered as a weakness that we did not assess synchronisation using the random placement of landmarks. However, we aimed to perform a methodological study. Random markers might affect synchronisation and we would recommend to assess it on the phantom before using it on a human fetus.

We have assessed synchronisation on a static fetus phantom with both MRI and real‐time US. Synchronisation and differences in measurements need to be tested on a live human fetus where fetal movements and tissue structures might affect the results.

## Conclusion

FI showed good synchronisation in measurements. BPD, HAP and VS were larger at MRI than US, likely a result of the way images are generated. Intra‐, inter‐observer differences between measurements were small. This can be important when reporting geometric measures from FI.

## Authorship declaration

I, Anastasija Arechvo, on behalf of all authors, declare that the authorship listing conforms with the journal’s authorship policy, and that all authors are in agreement with the content of the submitted manuscript.

## Funding

No funding information is provided.

## Conflict of Interest

None declared.

## Author Contributions

**Anastasija Arechvo:** Conceptualisation (equal); Formal analysis (equal); Investigation (equal); Methodology (equal); Project administration (equal); Software (equal); Visualisation (equal); Writing‐original draft (equal); Writing‐review & editing (equal). **Göran Lingman:** Conceptualisation (equal); Project administration (equal). **Lars Thurn:** Methodology (equal); Supervision (equal); Visualisation (equal). **Tomas Jansson:** Methodology (equal); Supervision (equal). **Ligita Jokubkiene:** Methodology (equal); Supervision (equal); Writing‐review & editing (equal).
